# Pathogen- and Host-Directed Anti-Inflammatory Activities of Macrolide Antibiotics

**DOI:** 10.1155/2012/584262

**Published:** 2012-06-21

**Authors:** Helen C. Steel, Annette J. Theron, Riana Cockeran, Ronald Anderson, Charles Feldman

**Affiliations:** ^1^Medical Research Council Unit for Inflammation and Immunity, Department of Immunology, Faculty of Health Sciences, University of Pretoria and Tshwane Academic Division of the National Health Laboratory Service, P.O. Box 2034, Pretoria 0001, South Africa; ^2^Division of Pulmonology, Department of Internal Medicine, Charlotte Maxeke Academic Hospital, Faculty of Health Sciences, University of the Witwatersrand, Johannesburg 2193, South Africa

## Abstract

Macrolide antibiotics possess several, beneficial, secondary properties which complement their primary antimicrobial activity. In addition to high levels of tissue penetration, which may counteract seemingly macrolide-resistant bacterial pathogens, these agents also possess anti-inflammatory properties, unrelated to their primary antimicrobial activity. Macrolides target cells of both the innate and adaptive immune systems, as well as structural cells, and are beneficial in controlling harmful inflammatory responses during acute and chronic bacterial infection. These secondary anti-inflammatory activities of macrolides appear to be particularly effective in attenuating neutrophil-mediated inflammation. This, in turn, may contribute to the usefulness of these agents in the treatment of acute and chronic inflammatory disorders of both microbial and nonmicrobial origin, predominantly of the airways. This paper is focused on the various mechanisms of macrolide-mediated anti-inflammatory activity which target both microbial pathogens and the cells of the innate and adaptive immune systems, with emphasis on their clinical relevance.

## 1. Introduction

**Figure 1:**
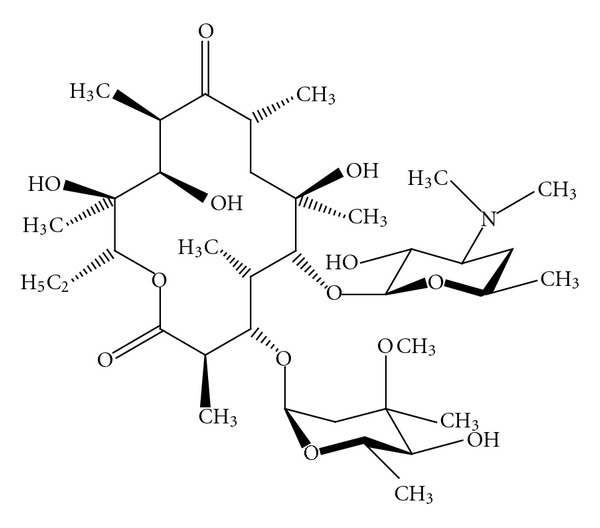
The molecular structure of erythromycin, the 14-membered prototype macrolide [1].

Macrolides, which are primarily antibiotics, belong to the polyketide group of natural products [1]. They derive their name from their characteristic structural features, a macrocyclic lactone ring to which various deoxy sugars, most commonly cladinose and desosamine, are attached [1]. The most important macrolide antibiotics are 14-, 15-, and 16-membered compounds. The molecular structure of the 14-membered erythromycin, the prototype macrolide, is shown in Figure 1. Drug delivery problems resulting from acid instability prompted the design of newer macrolides. These compounds include (i) clarithromycin, roxithromycin, dirithromycin, and the ketolides and fluoroketolides, all of which have a 14-membered ring structure; (ii) the 15-membered azithromycin; and (iii) the 16-membered agents spiramycin, rokitamycin, and josamycin.

Macrolide antibiotics are generally used to treat respiratory and soft tissue infections caused by Gram-positive bacteria. They are also active against rickettsiae, chlamydiae, and *Mycoplasma pneumoniae*, as well as some Gram-negative bacterial pathogens, including *Bacteroides fragilis, Bordetella pertussis, Campylobacter *species, *Haemophilus influenzae, Helicobacter pylori, Legionella pneumophila, Moxarella catarrhalis,* and *Neisseria* species. The more advanced macrolides, azithromycin, and clarithromycin, as well as the ketolides/fluoroketolides, have several distinct advantages over erythromycin. These include extended spectrum of activity, improved pharmacokinetics, pharmacodynamics and tolerability, and once-daily administration [2]. Azithromycin and to a lesser extent clarithromycin are noted for their high and prolonged concentrations at sites of infection, reaching tissue levels of 10–100-fold and 2–20-fold greater than serum concentrations, respectively [3–5]. Both agents are also concentrated intracellularly by alveolar macrophages, attaining levels of approximately 400-fold (clarithromycin) and 800-fold (azithromycin) above serum concentrations [3]. The ketolide, telithromycin, also has excellent penetration into bronchopulmonary tissues and macrophages, while macrolides and macrolide-like agents are also accumulated by polymorphonuclear leukocytes (PMNL), which, in turn, effect the active delivery of these agents to sites of bacterial infection [3, 6].

With respect to their mechanism of antimicrobial action, macrolides are inhibitors of bacterial protein synthesis. This is achieved by reversible binding of these agents to the P site of the 50S subunit of the bacterial ribosome [1]. The macrolide/ribosome interaction has several apparent consequences, all of which result in inhibition of bacterial protein synthesis. These are (i) interference with peptidyltransferase, preventing polypeptide chain elongation; (ii) inhibition of ribosomal translocation; and (iii) untimely detachment of peptidyl-tRNA from the ribosome [1, 7, 8]. Macrolides, ketolides, and fluoroketolides possess 1, 2, and 3 ribosomal binding, sites respectively [1]. Although predominantly bacteriostatic, the high tissue and macrophage/PMNL concentrations attained by macrolides and macrolide-like agents may favour bactericidal activity *in vivo.*

Notwithstanding their primary antimicrobial activity, macrolides, unlike most other classes of antibiotic, also possess beneficial anti-inflammatory properties. These latter effects are achieved by two distinct mechanisms. Firstly, as a consequence of their primary ribosomal-targeted mechanism of antimicrobial action, they inhibit the production of proinflammatory microbial toxins and other virulence factors. Surprisingly, this pathogen-directed mechanism of anti-inflammatory activity has also been described for a number of ostensibly macrolide-resistant bacterial pathogens as described hereinafter. Secondly, macrolides have been reported to possess secondary anti-inflammatory activities which target cells of the innate and adaptive immune systems as well as structural cells.

The remainder of this paper is devoted to a consideration of the anti-inflammatory activities of macrolides and their therapeutic relevance.

## 2. Pathogen-Targeted Anti-Inflammatory Activities of Macrolides

Antibiotics cooperate with host defences to eradicate microbial pathogens. In this setting, the antibiotic-exposed pathogens are weakened, increasing their vulnerability to the cellular and humoral defences of the host. While these antibiotic/host defence interactions are clearly beneficial, some antibiotics may trigger over-exuberant inflammatory responses with potentially harmful consequences for the infected host. These include cell-wall-targeted, bactericidal antibiotics, especially, beta-lactams, as well as fluoroquinolones, which initiate the release of proinflammatory intracellular toxins and cell-wall components from damaged, disintegrating bacteria. Examples of these are the pneumococcal toxin, pneumolysin, as well as cell-wall-derived lipopolysaccharides and lipoteichoic acids. These initiate exaggerated inflammatory responses by several mechanisms, including (i) interactions with Toll-like receptors and nucleotide- oligomerization- (NOD-) like receptors on/in immune and inflammatory cells, as well as epithelial cells; and (ii) activation of complement cascades [9–11]. The harmful, proinflammatory activities of beta-lactams and fluoroquinolones have been demonstrated in a number of studies, either by measuring the release of intracellular toxins following exposure of susceptible bacteria to these antimicrobial agents *in vitro* [12–18], or in animal models of experimental infection in which survival is correlated with the antimicrobial and proinflammatory potencies of antibiotics [19–22].

In contrast to beta-lactams and fluoroquinolones, antibiotics which inhibit bacterial protein synthesis, particularly macrolides and macrolide-like agents, prevent the release of proinflammatory protein toxins from both Gram-positive and Gram-negative bacteria, as well as the production of other virulence factors such as bacterial adhesins and biofilm. Consequently, the pathogen-targeted actions of macrolides have a much lesser propensity to trigger harmful inflammatory reactions than is the case with abruptly bactericidal agents, a contention which is supported by a considerable body of experimental evidence. This includes a number of *in vitro* studies which have demonstrated the inhibitory effects of macrolides and macrolide-like agents, often at subminimal inhibitory concentrations (MICs), on the production of proinflammatory/cytocidal bacterial toxins such as (i) pneumolysin by *Streptococcus pneumoniae* [23, 24], (ii) Panton-Valentine leukocidin and *α*-haemolysin by *Staphylococcus aureus* [12, 13], and (iii) shiga-like toxins by enterohaemorrhagic strains of *Escherichia coli* [14–18]. In contrast, exaggerated release of these toxins was observed when the bacteria were exposed to beta-lactams or fluoroquinolones [12–18, 25].

These findings have been confirmed in animal models of experimental infection. Spreer et al. in several studies using a rabbit model of experimental meningitis have reported that administration of the macrolide-like agent, clindamycin, as well as rifampicin, but not the beta-lactam, ceftriaxone, significantly reduced concentrations of pneumolysin in cerebrospinal fluid [19–21]. This was associated with an attenuated inflammatory response and decreased neuronal injury. More recently, others have investigated the effects of treatment with (i) ampicillin only, (ii) azithromycin or clindamycin only, or (iii) ampicillin in combination with either azithromycin or clindamycin on survival using a murine model of secondary, influenza-associated pneumococcal pneumonia [22]. The lowest survival rate in the antibiotic-treated animals was observed in mice treated with ampicillin only, while the highest rates were noted in those treated with azithromycin or clindamycin individually or in combination with ampicillin. Improved survival in the azithromycin/clindamycin-treated groups was associated with an attenuated inflammatory response in the airways characterized by decreases in both the numbers of inflammatory cells and concentrations of proinflammatory cytokines, as well as less severe histopathological changes [22].

In addition to the aforementioned effects of macrolides on dampening potentially harmful responses in the setting of acute bacterial infections caused by macrolide-susceptible pathogens, it is noteworthy that these agents have also been reported to inhibit the production of proinflammatory toxins by ostensibly macrolide-resistant pathogens. Notwithstanding the inhibitory effects of macrolides on the production of shiga toxins by *E. coli* mentioned previously, these agents have also been reported to inhibit the production of pneumolysin by macrolide-resistant strains of the pneumococcus both *in vitro *and* in vivo.* In an earlier study, Lagrou et al. reported that exposure of an *ermAM*-expressing, ribosomal methylase-producing, macrolide-resistant (MIC ≥ 256 *μ*g/mL) strain of *Streptococcus pneumoniae *to a sub-MIC concentration of erythromycin prevented the adherence of the bacteria to human nasal respiratory epithelial cells [26]. Although the growth of the bacteria was unaffected, exposure to erythromycin almost completely attenuated the production of pneumolysin, which was the probable cause of interference with bacterial adherence [26]. These findings were confirmed in a later study in which Fukuda et al. reported that both azithromycin and clarithromycin at concentrations of 1–4 *μ*g/mL inhibited the production of pneumolysin by *ermB *and* mefE/A *coexpressing, macrolide-resistant (MIC ≥ 256 *μ*g/mL) strains of the pneumococcus *in vitro* [27]. Administration of these agents to mice (40–200 mg/kg) experimentally infected with macrolide-resistant pneumococci was found to result in prolonged survival, which was associated with decreased concentrations of pneumolysin in the airways. Similar findings have been described by Anderson et al., who reported that exposure of an *ermB*-expressing, macrolide-resistant strain of *S. pneumoniae*(MIC ≥ 256 *μ*g/mL) to a range of macrolides and macrolide-like agents (0.5 *μ*g · mL) resulted in significant attenuation of the production of pneumolysin, while amoxicillin, ceftriaxone, ciprofloxacin, doxycycline, and tobramycin were ineffective [23, 24].

More recently, Cockeran et al. have attempted to identify the molecular basis of the inhibitory effects of macrolides on the production of pneumolysin by macrolide-resistant strains of the pneumococcus [28]. They observed that exposure of 8 different *ermB*-expressing, macrolide-resistant strains (each with an MIC value of >256 *μ*g/mL) to clarithromycin resulted in significant prolongation of the lag phase of bacterial growth (4.9–12.2 hours in comparison with 1.2–4.9 hours for non-exposed bacteria). Although rapid induction of the *ermB* gene was evident, according to a 4-fold increase in mRNA within 15 minutes of exposure to the antibiotic, synthesis of ribosomal methylase is probably hindered because of binding of clarithromycin to the peptide exit tunnel of the large ribosomal subunit, blocking peptide chain elongation [28]. The consequence is transient susceptibility due to slow acquisition of the full resistance phenotype. 

Additional mechanisms which have been reported to underpin the efficacy of macrolides in murine models of experimental infection include high levels of intracellular accumulation of these agents by phagocytes and epithelial cells as well as their beneficial, secondary anti-inflammatory properties described hereinafter [29, 30].

### 2.1. Macrolides and *Pseudomonas aeruginosa*

*Pseudomonas aeruginosa* is a persistent opportunistic pathogen which colonizes the airways of immunocompromised individuals causing a chronic, ineffectual inflammatory response. This in turn results in inflammation-mediated tissue damage and pulmonary dysfunction and is particularly serious in patients with cystic fibrosis. Although macrolides do not affect the growth of *P. aeruginosa*, they are nevertheless protective by inhibiting the production of persistence-promoting and proinflammatory virulence factors. These include (i) proadhesive type IV pili, (ii) tissue-damaging pseudomonal elastase, (iii) proinflammatory rhamnolipid, and (iv) alginate and biofilm [31–34]. Alginate is an exopolysaccharide which functions as an antiphagocytic capsule, while biofilm is a self-generated, extracellular polymer matrix in which the pathogen is insulated against both antibiotics and the cellular and humoral defences of the host.

These *P. aeruginosa*-directed anti-infective, anti-inflammatory activities of macrolides, including erythromycin, clarithromycin, and azithromycin, appear to target quorum sensing in *P. aeruginosa*. Quorum sensing is a mechanism of microbial intercellular communication, utilising diffusible signalling molecules known as autoinducers, which enable bacteria to detect and regulate their population density and to upregulate virulence [35]. Gram-negative bacteria most commonly utilize type I family autoinducers known as N-acylated-L-homoserine lactones as their primary mediators of quorum sensing [35]. Both azithromycin and clarithromycin have been reported to inhibit the production of this class of autoinducers by *P. aeruginosa* [31, 36, 37]. Importantly, these effects were evident at sub-MIC concentrations of both macrolides, which in the case of azithromycin was 2 *μ*g/mL [36]. In the case of biofilm formation, the quality of biofilm, as opposed to initiation of synthesis, appeared to be impaired by the macrolides, resulting in altered architecture, structure, and density, favouring the penetration of antibiotics [36, 37]. The pathogen-directed anti-inflammatory activities of macrolides are summarised in Table 1.

As a strategy to counter *P. aeruginosa* in particular, the aforementioned antimicrobial/anti-inflammatory activities of macrolides are of proven benefit in the long-term therapy of cystic fibrosis [38], as well as the other chronic inflammatory disorders of the airways described hereinafter. However, the benefits of long-term administration of macrolides must be balanced against the potential risks, which include development of macrolide resistance, and, of particular concern, increased susceptibility to infection with nontuberculosus mycobacteria as a consequence of interference with lysosomal acidification [39].

## 3. Effects of Macrolides on Innate and Adaptive Immune Mechanisms

In addition to pathogen-directed anti-inflammatory activity, macrolides have also been reported to inhibit the proinflammatory activities of cells of both the innate and adaptive immune systems.

### 3.1. Innate Immunity

In the setting of innate immunity, the predominant anti-inflammatory activity of macrolides appears to be achieved via the modulation of the proinflammatory activities of neutrophils, in particular, inhibition of the production of the potent neutrophil activator and chemoattractant, IL-8 [40, 41]. Increased IL-8 in sputum and bronchoalveolar lavage is associated with severity of chronic inflammatory diseases such as cystic fibrosis (CF) and diffuse panbronchiolitis (DPB) [41–44]. Azithromycin, erythromycin, and clarithromycin have been shown to attenuate the production and secretion of IL-8 by airway smooth muscle cells, alveolar macrophages, and human gingival fibroblasts [40, 45, 46], as well as other cytokines such as (i) IL-1*α* and IL-2 by murine macrophages and splenocytes, respectively; (ii) IL-1*β*, GM-CSF, TNF-*α*, and MCP-1 by macrophages; and (iii) IL-1*β*, IL-6 and TNF-*α* from peripheral blood monocytes [47–53]. This is thought to result from the suppression of nuclear translocation of several transcription factors [54] by the macrolides, specifically nuclear factor- (NF-) *κ*B, activator-protein- (AP-) 1, and specificity protein 1 in various types of inflammatory and structural cells [40, 54–60]. Inhibition of intracellular signalling via the extracellular signal-regulated kinase 1 and 2 (ERK 1/2) and p38 mitogen-activated protein kinase (MAPK) pathways are thought to mediate the downregulation of NF-*κ*-B, AP-1, and specificity protein 1 in response to clarithromycin [56, 57, 61–64]. In addition, azithromycin has been shown to attenuate the LPS/IFN-*γ*-mediated induction of IL-12p40, probably by the inhibition of the binding of AP-1, nuclear factor of activated T cells (NFAT), and interferon consensus sequence binding protein (ICSBP) to the DNA binding site of the IL-12p40 promoter [65]. This may also prove to be an important mechanism for regulating the anti-inflammatory effects of azithromycin in macrophages.

Interestingly, the ability of macrolide antibiotics to modulate cytokine expression by human neutrophils and their ability to decrease or increase cytokines is thought to depend on the presence or absence of bacteria [66, 67]. Clarithromycin was shown to inhibit the production of IL-6 and TNF-*α* by neutrophils primed with lipopolysaccharide (LPS), while increasing their expression when bacteria were present [67]. Shinkai et al. reported that clarithromycin initially increased IL-8 secretion by bronchial epithelial cells via ERK signalling but later inhibited ERK signalling leading to reduction (normalisation) in secretion of the chemokine. It is suggested that immunomodulation occurs, in part, by sequential cycles of ERK 1/2 inhibition and activation [60, 63]. This modulation of ERK 1/2 and transcription factors is consistent and unrelated to the antimicrobial properties of macrolides.

Notwithstanding interference with the production of IL-8 by monocytes/macrophages and various types of structural cells, several other mechanisms have been described by which macrolides inhibit neutrophil migration. These include (i) decreased synthesis and expression of the endothelial adhesion molecules ICAM-1 and VCAM-1, possibly as a consequence of decreased synthesis of IL-1*β* and TNF-*α* by tissue macrophages and other cell types [68, 69], (ii) interference with the expression of *β*2-integrins on activated neutrophils [69], (iii) decreased synthesis of leukotriene B_4_, a potent neutrophil chemoattractant, possibly as a secondary consequence of inhibitory effects on cytokines/chemokines [70], and (iv) interference with the synthesis and release of the matrix- metalloproteinases- (MMP-), 2, 7, and 9 from nasal polyp fibroblasts, as well as neutrophils, via antagonism of activation of NF-*κ*B and AP-1 [71–73]. MMPs facilitate neutrophil migration.

In addition, macrolides may also interfere with signalling mechanisms initiated by activation of Toll-like receptors (TLRs). TLRs play a key role in innate host defence against viral and microbial pathogens by promoting the release of the neutrophil-mobilizing cytokines, IL-8, and TNF-*α*, from tissue macrophages and epithelial cells in particular. Treatment of monocyte-derived dendritic cells with erythromycin resulted in up-regulation of TLR2, down-regulation of TLR3, and no effect on expression of TLR4 [74]. However, clarithromycin has been reported to downregulate the expression of TLR4 on monocytes infected with *Helicobacter pylori *[75]. These results indicate that macrolides may selectively downregulate inflammatory responses which result from the interaction of viruses and Gram-negative bacteria with TLR3 and TLR4, respectively, while maintaining the interaction of Gram-positive bacteria with TLR2 [75].

Other anti-inflammatory interactions of macrolides with neutrophils include interference with the generation of reactive oxygen species (ROS) by these cells [76]. Although several mechanisms may exist, membrane-stabilizing activity has been proposed to underpin these effects by neutralizing the sensitizing actions of bioactive phospholipids such as lysophosphatidylcholine, platelet-activating factor (PAF), and lysoPAF on the membrane-associated, superoxide-generating complex of neutrophils, NADPH oxidase [77]. Macrolides have also been reported to induce phospholipidosis in eukaryotic cells, the magnitude of which appears to correlate with anti-inflammatory activity [78, 79]. Macrolides have also been reported to suppress the production of another type of ROS, nitric oxide, by activated macrophages, presumably by interfering with the induction of inducible nitric oxide synthase via antagonism of NF-*κ*B [80, 81]. The anti-inflammatory interactions of macrolides with the cells of the innate immune system are summarised in Table 2.

In addition to their effects on neutrophils and macrophages, macrolides, as alluded to what is mentioned before, can also downregulate the proinflammatory activities of structural cells, especially epithelial cells. Airway epithelial cells not only provide a mechanical barrier to inhaled micro-organisms but are also involved in the direct killing of microbial pathogens, as well as in activating other cells of the innate immune system [63]. The upper and lower respiratory tracts are lined by a highly specialised ciliated columnar epithelium which, together with the mucous layer covering these cells, constitute the mucociliary escalator which functions to keep the lower respiratory tract pathogen-free [82]. Macrolides have been shown to stimulate ciliary beat frequency and improve mucociliary clearance [83, 84]. Moreover, erythromycin, azithromycin, clarithromycin, and roxithromycin have been shown to inhibit chemotaxis and infiltration of neutrophils into the airways and subsequently suppress the synthesis and release of mucus by inhibiting *muc5ac* gene expression [68, 85–87]. Clarithromycin inhibits *muc5ac* gene expression, while azithromycin has been shown to inhibit *muc5ac* production in an ERK 1/2-dependent manner [68, 88]. Macrolides may also decrease sputum production by inhibiting chloride secretion [68]. In addition to these anti-inflammatory effects of macrolides on epithelial cells, these agents have also been reported to protect ciliated respiratory epithelium against the damaging effects of host-derived bioactive phospholipids [89].

### 3.2. Adaptive Immunity

Although lymphocytes are essential for adaptive immune responses to pathogens, they may also play a harmful role in inflammatory conditions such as autoimmunity and bronchial asthma. Several studies have described the anti-inflammatory effects of macrolides on lymphocytes, particularly T-lymphocytes. These include inhibition of proliferation of (i) Jurkat T cells treated with erythromycin and its non-antibacterial derivatives [90]; (ii) CD4 T cells, when clarithromycin- and roxithromycin-treated and untreated dendritic cells were used as antigen presenting cells [91]; (iii) peripheral blood mononuclear cells treated with azithromycin, clarithromycin, and roxithromycin and activated with concanavalin-A or toxic shock syndrome toxin-1 [92]; and (iv) T cells from house dust mite allergen-sensitive bronchial asthma patients treated with roxithromycin and stimulated with mite antigen [93]. In contrast, cystic fibrosis patients who were treated with clarithromycin (250 mg/day) and followed for a year showed a sustained increase in the *ex vivo* proliferative responses of peripheral blood lymphocytes activated with the T-cell mitogen, phytohemagglutinin [94], possibly reflecting transient inhibitory effects of the macrolides.

The effects of macrolides on cytokine production by T-lymphocytes have also been described in a number of studies. In their study, Pukhalsky et al. reported reversal of the serum IFN-*γ*/IL-4 ratio in cystic fibrosis patients treated with clarithromycin, compatible with a potentially beneficial elevation in the Th1/Th2 ratio [94]. Others also reported that roxithromycin and clarithromycin increased the Th1/Th2 ratio by decreasing production of IL-4 and IL-5, without affecting IL-2 and IFN-*γ* levels in several experimental systems, including (i) T cells isolated from the blood of healthy and allergic rhinitis subjects [95], (ii) house dust mite antigen-induced responses of peripheral blood lymphocytes of mite-sensitive bronchial asthma patients [93], and (iii) mononuclear leucocytes, isolated from the blood of healthy donors and stimulated with phorbol 12-myristate 13-acetate (PMA) and ionomycin [96]. In contrast to these findings, Park et al. reported that patients with diffuse panbronchiolitis, receiving long-term treatment with erythromycin, showed decreased levels of IL-2 and IFN-*γ*, in the setting of increased levels of IL-4, IL-5, and IL-13 in the bronchoalveolar lavage fluid, suggesting a shift from Th1 to Th2 cytokine production following treatment with the macrolide [97]. Inhibition of the production of cytokines by T-lymphocytes by macrolides was also demonstrated in various other studies [91, 92, 98].

T-cell chemotaxis and apoptosis are also affected by treatment with macrolides. Th1, Th2, but not T regulatory cells, treated with roxithromycin, elicited reduced chemotactic responses to the chemokines IP10 (IFN-*γ*-inducible protein 10) and TARC (thymus- and activation-regulated chemokine) [99]. In addition, erythromycin, clarithromycin, azithromycin, and josamycin have been reported to induce apoptosis in lymphocytes, potentially reducing the number of lymphocytes in the lungs of patients with chronic respiratory tract diseases [90, 100–102].

Apart from effects on T cells, macrolides also appear to affect B-lymphocytes, specifically the expression of co-stimulatory molecules. Asano et al. reported that treatment of B-lymphocytes isolated from BALB/c mice spleens with roxithromycin (5.0 *μ*g/mL) resulted in significant suppression of the expression of the costimulatory molecules, CD40, CD80, and CD86, induced by antigenic stimulation *in vitro* [103]. The anti-inflammatory interactions of macrolides with cells of the adaptive immune system are shown in Table 3.

From a mechanistic perspective, these immunomodulatory activities of macrolides appear to be polymodal. Nonetheless, the weight of evidence favours inhibition of extracellular signal-regulated kinase 1/2 (ERK 1/2) phosphorylation and NF-*κ*B activation as being the predominant mechanisms [104, 105].

## 4. Immunolides

The clinical efficacy of macrolides in the therapy of apparently nonmicrobial chronic inflammatory diseases of the airways has triggered the design and development of a novel class of macrolides, known as immunolides, which are attenuated with respect to antimicrobial activity in the setting of retention of anti-inflammatory properties [56, 106]. These include (i) 9- (*S*)-dihydroerythromycin derivatives which have been demonstrated to possess impressive anti-inflammatory activity in a murine model of phorbol ester-induced ear oedema [107], and (ii) more recently, the EM900 series of novel 12-membered, erythromycin-A-derived nonantibiotic macrolides [108]. EM900 was found to promote monocyte to macrophage differentiation, while suppressing activation of NF-*κ*B and IL-1*β*, IL-8, and TNF-*α* gene expression in a human airway epithelial cell line (A549) activated with IL-1*β*, as well as mucin (*muc5ac*) gene expression by HM3-muc5ac cells [58]. Although promising, the development of immunolides remains in the preclinical stages. Nonetheless, it is our belief that it is the combination of antimicrobial and immunomodulatory properties, as described previously, that is most likely to confer optimum anti-inflammatory activity on the macrolide/azalide/ketolide group of antibiotics.

## 5. Clinical Conditions for Which Macrolides Are Used Primarily for Their Anti-Inflammatory, Immunomodulatory Properties

Many of the medical conditions for which macrolides are used primarily for their alternative properties, rather than their antimicrobial activity, are chronic disorders of the airway, of both the upper and lower respiratory tract, in which inflammation plays a major pathogenic role [109–112]. While in some of these disorders, such as DPB and CF, evidence for macrolide use is well accepted so that these agents have been included internationally as part of the standard of care, in other conditions, however, the evidence is somewhat less well established, and here these agents are used much more selectively, and particularly in cases that are not responding adequately to more standard therapy. The alternative mechanisms by which macrolides appear to have benefit mostly relate to the cytoprotective effects of these agents on human-ciliated epithelium, their anti-inflammatory, immunomodulatory activity, and their inhibitory activity against quorum sensing mechanisms of a number of important respiratory tract pathogens as mentioned previously [69, 104, 110, 111, 113–116].  Table 4 indicates some of the more common conditions for which macrolide use has been considered. Hereinafter are brief summaries of the evidence for the possible benefits and/or roles of macrolides in various medical conditions, based on an overview of appropriate scientific studies and reviews.

### 5.1. Diffuse Panbronchiolitis (DPB)

DPB is a chronic inflammatory disorder of the airway occurring in many population groups, but being most common among individuals of Japanese origin [109–112]. The major presentation is with cough, sputum production, and progressive shortness of breath, and patients very frequently become colonised with pseudomonal isolates. Without any treatment the outcome of DPB is dismal. Chronic low-dose macrolide therapy is the treatment of choice and has had a major positive impact on the natural history of this condition [109–112, 117–130].

### 5.2. Cystic Fibrosis (CF)

CF is an autosomally recessive inherited disorder occurring predominantly in Caucasian populations in which abnormalities in epithelial cell ion transport occur as a consequence of defects in the CF transmembrane regulator, resulting in increased sputum viscosity, stasis of secretions, airway infection and inflammation, and progressive bronchiectasis. A myriad of studies has been conducted in the past 10 years evaluating the possible role of long-term macrolide therapy in this condition [94, 110–112, 131–153]. When evaluating these as a whole there is clear-cut evidence that long-term macrolide treatment has benefit with regard to clinically relevant end-points in patients with CF and macrolide therapy features prominently in guidelines for its management, particularly in those cases infected with *Pseudomonas aeruginosa* who have associated deterioration in lung function. It is interesting to note that the mechanisms of action of macrolides in such CF patients appear to relate not only to their antineutrophil, anti-inflammatory activities but also to their detrimental effects on the biology of *P. aeruginosa*, which have been well characterised [94, 110–112, 130–153].

### 5.3. Non-CF Bronchiectasis

Bronchiectasis is a condition most commonly occurring as a consequence of chronic airway infection and inflammation. In this disorder, airway obstruction mainly associated with bacterial infection, and its associated airway inflammation, leads to a “vicious circle” of chronic infection and inflammation with progressive damage to the ciliated epithelium lining the airways and subsequently its underlying structures. The condition is associated not only with airway disease punctuated by recurrent acute infective exacerbations but also with chronic systemic debility leading to considerable morbidity and even mortality. Since chronic airway inflammation is central to its pathogenesis and few other therapies have been shown to alter the natural course of the condition, it is not surprising that anti-inflammatory therapies of all sorts have been tried in this condition, of which the macrolides appear to be the most promising [36, 154–177]. Interest in macrolide use for non-CF bronchiectasis was developed following their successful use in patients with CF. Beneficial effects of long-term macrolide use for non-CF bronchiectasis have been found in small clinical trials. In most of these studies there was clear evidence of a decrease in sputum volume and, in some, a decrease in exacerbation frequency. Furthermore, in a small number in which this was tested there was an improvement in lung function parameters or a decrease in airway hyperreactivity. The common recommendation for this condition is to try macrolide therapy in selected cases for 3–6 months and to discontinue treatment if there is no clear evidence of benefit to the patient in terms of improvement in quality of life or reduction in exacerbation frequency.

### 5.4. Bronchiolitis Obliterans (BOs)

BO is one of the manifestations of chronic rejection following lung or bone marrow transplant and is a major cause of limited survival and death in lung transplant recipients. Although the exact pathogenesis has still to be unravelled, it appears to result as a consequence of repeated insults to the airways. More recently there has been considerable interest in using macrolides for this serious condition for which other therapies have been rather disappointing or are associated with considerable side-effects [178–189]. Studies have been undertaken to investigate not only the effects of macrolides as therapy for this condition but also, more recently, its prevention. In reviewing the various therapeutic studies, it has been said that there are differences in the clinical spectrum and macrolide response of patients with BO and that those cases associated with a predominantly neutrophilic pathogenesis are macrolide responsive, while those associated with a predominantly fibroproliferative response (so-called traditional BO) are not.

### 5.5. Chronic Obstructive Pulmonary Disease (COPD)

In more recent definitions of COPD, due recognition is given to the fact that in this condition there is an abnormal inflammatory process in the airways, which, although initially is most commonly associated with cigarette smoking, at some stage becomes self-perpetuating and contributes to the progressive deterioration that may be seen in patients with COPD, even in those that quit smoking. While macrolides may be used for the antibiotic management of acute exacerbations of COPD, studies have also been conducted wherein these agents are used for their anti-inflammatory, immunomodulatory activities and their effects on mucus secretion. In most of these studies a reduction in sputum production, as well as improvement in the quality of the sputum, has been noted, while in some an improvement in quality of life, various clinical end-points, and occasionally in lung function parameters has been seen. Importantly, some studies have suggested that macrolide therapy may alter the course of COPD by reducing both the number and the duration of acute exacerbations [68, 109, 190–199].

### 5.6. Asthma

It has been recognised for a number of years that asthma is a chronic inflammatory disorder of the airways, the inflammation being mediated by a variety of cells and mediators which are responsible for the manifestations including the symptoms, the lung function abnormalities, and the airway hyperresponsiveness. Therapy is therefore primarily with anti-inflammatory agents, particularly inhaled corticosteroids, but a number of the other drugs used in asthma treatment have also been recognised to have anti-inflammatory activity. While much of the airway inflammation may be driven by allergic/atopic responses, it has also been suggested that chronic lower respiratory tract infection with *Mycoplasma pneumoniae *and *Chlamydia pneumoniae*, both microorganisms that are responsive to macrolide therapy, may initiate airway inflammation and asthma and is therefore potentially amenable to macrolide therapy. All of these considerations provide the rationale for the use of macrolides in asthma, in the hope of achieving more effective asthma control. Although a number of studies have been undertaken over more recent years using different macrolides, with some showing modest benefits, the overall data suggests that there is no role for long-term macrolide therapy in asthma, although such treatment may be of benefit in some subgroups of patients, such as those described previously [200–214].

### 5.7. Pneumonia

Antibiotic therapy in patients with pneumonia is short course, aimed at treating the infection and eradicating the microorganism. However, there is still considerable ongoing debate as to what antibiotic regimen constitutes optimal therapy in hospitalised cases with community-acquired pneumonia (CAP), including those that require intensive care unit (ICU) admission. A myriad of studies in more severely ill-hospitalised patients with CAP has suggested that the outcome is improved by using combination antibiotic therapy, most commonly with the addition of a macrolide to standard beta-lactam therapy [215–226]. This understanding needs to be counterbalanced by additional studies suggesting that the outcome is similar when comparing fluoroquinolone monotherapy to the beta-lactam/macrolide combination in noncritically ill-hospitalised patients [227–229]. Thus for cases not in the ICU, most guidelines recommend either option, whereas in ICU patients, combination therapy is always recommended irrespective of which of these agents is used. Interestingly, in one study in intubated patients in the ICU, the outcome was better with the use of the macrolide rather than the fluoroquinolone combination [226]. The reason that combination therapy with macrolides is associated with an improved outcome in patients with CAP is uncertain and may be multifactorial; however, many believe that it may relate to the anti-inflammatory immunomodulatory effects of these agents [229]. Two recent studies appear to support this contention [230, 231]. In the first study, macrolide use was associated with decreased mortality in patients with CAP and severe sepsis even when the infection was due to macrolide-resistant pathogens. Furthermore, a placebo-controlled, randomised, clinical trial, undertaken to investigate whether patients with sepsis and ventilator-associated pneumonia (VAP), predominantly due to Gram-negative pathogens, had improved outcome when a macrolide was added to standard antibiotic therapy, demonstrated that clarithromycin accelerated the resolution of VAP and the weaning from mechanical ventilation and delayed death in those that ultimately died of sepsis. In addition, in a very recent review of the literature, Kovaleva, et al. concluded that macrolides appear to attenuate the inflammatory response during CAP [232]. In support of this contention, Walkey and Weiner have reported, also very recently, that patients with acute lung injury (ALI), predominantly associated with pneumonia, who were treated with macrolides, had a significantly lower 180-day mortality and shorter time to successful discontinuation of mechanical ventilation relative to those patients treated with fluoroquinolones or cephalosporins [233]. 

### 5.8. Upper Respiratory Tract Disorders

A number of studies have also been undertaken investigating the use of macrolides in upper airway conditions, such as chronic rhinosinusitis, and appear to show promise [234–244]. Such studies clearly suffer from the methodological issues discussed hereinafter and need to be repeated in appropriate fashion before conclusions can be drawn about the value of macrolides and their use in upper airway diseases, although recommendations for macrolide use do appear in many of the international guidelines on rhinosinusitis management, in certain circumstances. As in many of the conditions already discussed, these potential benefits are thought to relate to the anti-inflammatory, immunomodulatory activity of macrolides and their effects on the virulence of and tissue damage caused by the chronic colonising bacteria [234–244]. 

## 6. Conclusions

It is clear from the various studies that macrolides have a clear-cut role in conditions such as DPB and CF, and possibly additional beneficial effects on morbidity, and possibly even mortality, in various other airway disorders. Furthermore, additional studies have also uncovered potential beneficial effects in various disorders unrelated to the airway. Many of these studies suffer from the fact that they are limited in terms of size, patient numbers, and length of treatment and follow-up. It is therefore clear that in many of these conditions further studies are needed in order to clarify such questions as in which patients these agents should be used, which macrolide drugs is/are best, what dosing schedules are appropriate, for how long should treatment be continued, and what are the long-term side-effects?

## Figures and Tables

**Table 1:** Targets of the pathogen-directed anti-inflammatory activities of macrolide antibiotics.

(i) Synthesis and release of proinflammatory toxins and virulence factors	
(ii) Quorum sensing	
(iii) Biofilm formation	

**Table 2:** Anti-inflammatory effects of macrolides on phagocytes and structural cells.

Cellular target	Altered function	Mechanisms
Neutrophils	↓ Migration	Interference with (i) production of IL-8 and TNF-*α* by macrophages and structural cells, (ii) decreased expression of adhesion molecules on vascular endothelium and neutrophils, and (iii)↓ production/release of MMPs by fibroblasts and neutrophils
	↓ production of ROS	Interference with NADPH oxidase, possibly by antagonizing the sensitizing actions of bioactive phospholipids
Macrophages	↓ cytokine production (IL-1*β*, IL-6, IL-8, TNF-*α*)	Interference with intracellular signalling mechanisms and transcription factor activation, resulting in suppression of gene expression
	↓ decreased NO production	As above, resulting in decreased expression of the gene encoding iNOS
Airway epithelial cells, fibroblasts, smooth muscle cells	↓ cytokine production (IL-8, TNF-*α*)	As above

**Table 3:** The anti-inflammatory effects of macrolides on T- and B-lymphocytes.

Cellular target	Altered function	Mechanisms
T-lymphocytes	↓ Proliferation	Interference with (i) expression of NF*κ*B,(ii) cellular JNK & ERK activity, and (iii) IFN-*γ* levels (enhancement may contribute to anti-proliferative activity)
T-lymphocytes	↓ Cytokines of either Th1 (IL-2, TNF-*α*, IFN-*γ*), Th2 (IL-4, IL-5, IL-10, IL-13) or both cell types	Interference with cellular JNK and ERK activity
T-lymphocytes	↓ Chemotaxis	Interference with F-actin polymerization and Ca^2+^ influx
T-lymphocytes	↑ Apoptosis	Interference with (i) NF-*κ*B activity,(ii) Bcl-xL expression, and (iii) Fas-Fas ligand pathway
B-lymphocytes	↓ Costimulatory molecules (CD40, CD80, CD86)	—

Abbreviations: NF-*κ*B: nuclear factor kappa-light-chain-enhancer of activated B cells; JNK: c-Jun N-terminal kinases; ERK: extracellular-signal-regulated kinases; Bcl-xL: B-cell lymphoma-extra large.

**Table 4:** Conditions for which macrolide use may be beneficial, primarily as a result of their anti-inflammatory, immunomodulatory activity.

(i) Diffuse panbronchiolitis
(ii) Cystic fibrosis (CF)
(iii) Non-CF bronchiectasis
(iv) Bronchiolitis obliterans
(v) Chronic obstructive pulmonary disease
(vi) Asthma
(vii) Pneumonia
